# Multicenter Evaluation of BioFire FilmArray Respiratory Panel 2 for Detection of Viruses and Bacteria in Nasopharyngeal Swab Samples

**DOI:** 10.1128/JCM.01945-17

**Published:** 2018-05-25

**Authors:** Amy L. Leber, Kathy Everhart, Judy A. Daly, Aubrey Hopper, Amanda Harrington, Paul Schreckenberger, Kathleen McKinley, Matthew Jones, Kristen Holmberg, Bart Kensinger

**Affiliations:** a Nationwide Children's Hospital, Columbus, Ohio, USA; b Primary Children's Medical Center, Salt Lake City, Utah, USA; c Loyola University Medical Center, Chicago, Illinois, USA; d BioFire Diagnostics, LLC, Salt Lake City, Utah, USA; Memorial Sloan Kettering Cancer Center

**Keywords:** PCR, respiratory pathogens, syndromic testing

## Abstract

The FilmArray Respiratory Panel 2 (RP2) is a multiplex *in vitro* diagnostic test for the simultaneous and rapid (∼45-min) detection of 22 pathogens directly from nasopharyngeal swab (NPS) samples. It contains updated (and in some instances redesigned) assays that improve upon the FilmArray Respiratory Panel (RP; version 1.7), with a faster run time. The organisms identified are adenovirus, coronavirus 229E, coronavirus HKU1, coronavirus NL63, coronavirus OC43, human metapneumovirus, human rhinovirus/enterovirus, influenza virus A, influenza virus A H1, influenza virus A H1-2009, influenza virus A H3, influenza virus B, parainfluenza virus 1, parainfluenza virus 2, parainfluenza virus 3, parainfluenza virus 4, respiratory syncytial virus, Bordetella pertussis, Chlamydia pneumoniae, and Mycoplasma pneumoniae. Two new targets are included in the FilmArray RP2: Middle East respiratory syndrome coronavirus and Bordetella parapertussis. This study provides data from a multicenter evaluation of 1,612 prospectively collected NPS samples, with performance compared to that of the FilmArray RP or PCR and sequencing. The overall percent agreement between the FilmArray RP2 and the comparator testing was 99.2%. The RP2 demonstrated a positive percent agreement of 91.7% or greater for detection of all but three analytes: coronavirus OC43, B. parapertussis, and B. pertussis. The FilmArray RP2 also demonstrated a negative percent agreement of ≥93.8% for all analytes. Of note, the adenovirus assay detects all genotypes, with a demonstrated increase in sensitivity. The FilmArray RP2 represents a significant improvement over the FilmArray RP, with a substantially shorter run time that could aid in the diagnosis of respiratory infections in a variety of clinical scenarios.

## INTRODUCTION

Upper respiratory infections are common and contribute significantly to morbidity and mortality. They are also one of the leading reasons for health care visits, thus resulting in significant health care costs (1, 2). Because the symptoms related to infections with many of the causative agents are very similar, definitive diagnosis requires laboratory testing. Toward that end, the concept of syndromic testing has been widely adopted, with testing for multiple agents of respiratory infection at the same time with a single test. By using these syndromic diagnostics, proper antimicrobial stewardship may be better achieved by allowing antimicrobial or antiviral therapy to be given in a timely and appropriate manner (3, 4). Most importantly, it may prevent the unnecessary use of antibiotics in the face of a viral diagnosis. Additionally, studies have demonstrated that rapid diagnosis of respiratory infections can lead to decreased length of stay, better antimicrobial stewardship, and better patient cohorting to prevent nosocomial infections (3, 5–8).

The FilmArray Respiratory Panel (RP) was first introduced as a syndromic multiplex molecular test in 2011 for the detection of 15 viruses; additional viral analytes and bacteria were made available with a software update in 2012 after FDA clearance for these new indications. Adenovirus inclusivity was improved with the addition of new primers after an additional FDA clearance in 2013 (version 1.7 [v1.7]). All FilmArray RP references henceforth in this article are to the current commercially available version of the device as of the publication of this paper, i.e., the FilmArray Respiratory Panel v1.7.

In order to ensure that a molecular diagnostic assay remains clinically relevant, and particularly for syndromic assays, it is important to periodically update the test to incorporate new sequence information and to accommodate emerging or previously unrecognized strains or pathogens. To this end, BioFire Diagnostics has updated the FilmArray RP product again by adding new assays to broaden the test's detection capabilities (particularly for adenoviruses), modifying a subset of assays to reflect newly available genetic sequences of currently included analytes, improving chemistry to enhance sensitivity overall, and for the inclusion of new analytes. The new test also has a decreased run time (∼45 min versus ∼65 min). The organisms detected by the FilmArray RP2 include all of those identified by the FilmArray RP: adenovirus, coronavirus 229E (CoV-229E), coronavirus HKU1 (CoV-HKU1), coronavirus NL63 (CoV-NL63), coronavirus OC43 (CoV-OC43), human metapneumovirus (hMPV), human rhinovirus/enterovirus (HRV/EV), influenza virus A (FluA), influenza virus A H1 (FluA H1), influenza virus A H1-2009 (FluA H1-2009), influenza virus A H3 (FluA H3), influenza virus B (FluB), parainfluenza virus 1 (PIV1), parainfluenza virus 2 (PIV2), parainfluenza virus 3 (PIV3), parainfluenza virus 4 (PIV4), respiratory syncytial virus (RSV), Bordetella pertussis (detection of *ptxP*), Chlamydia pneumoniae (previously named Chlamydophila pneumoniae), and Mycoplasma pneumoniae. Two new targets are included: Middle East respiratory syndrome coronavirus (MERS-CoV) and Bordetella parapertussis (detection of IS*1001*). Note that results for MERS-CoV are masked in the FilmArray RP2 product that is FDA cleared for the U.S. market. This analyte is reported in the FilmArray Respiratory Panel 2*plus* (RP2*plus*) product, which is sold outside the U.S. for testing individuals demonstrating signs/symptoms of respiratory infection and has been cleared by the U.S. FDA, with a modified intended use to aid in the differential diagnosis of MERS-CoV infections only in cases meeting MERS-CoV clinical and/or epidemiological criteria. The FilmArray RP2 is identical to the current FilmArray RP with respect to specimen type, handling, testing workflow, pouch controls, and analysis software.

In this study, data are presented for a prospective multicenter clinical evaluation of the performance of the FilmArray RP2 in residual nasopharyngeal swab (NPS) specimens collected in viral transport medium (VTM). Performance is compared to that of the FilmArray RP for 20 of 22 analytes (all those in common between the two tests) as well as that of PCR followed by bidirectional sequencing for B. parapertussis. MERS-CoV was not circulating in the United States during the time of the study; therefore, all specimens were assumed to be negative, and no comparator testing was performed for this analyte.

## MATERIALS AND METHODS

### Clinical specimens.

The study was conducted at three geographically distinct U.S. sites (Nationwide Children's Hospital [NCH], Columbus, OH; Loyola University Medical Center, Maywood, IL; Primary Children's Hospital, Salt Lake City, UT) over a period of approximately 6 months (January to March and September to November 2016). Between January and March 2016, specimens were collected and immediately frozen for later testing. Between September and November 2016, specimens were collected and tested fresh. Specimens meeting the following inclusion criteria were selected: the specimen was an NPS collected in VTM with adequate residual volume (≥1.5 ml), the specimen was tested with the FilmArray RP as the standard of care (SOC), and the specimen was held at room temperature for less than or equal to 4 h or at 4°C for less than or equal to 3 days before enrollment. A waiver of the informed consent requirement was obtained from the institutional review boards at each study site for the use of residual NPS specimens. Clinical and demographic data were collected, including hospitalization status at the time of specimen collection, the results of the clinician-ordered SOC FilmArray RP test, the date of specimen collection, the subject sex, and the subject age at time of collection.

### FilmArray RP2 testing.

Approximately 300 μl of specimen was subjected to FilmArray RP2 testing according to the manufacturer's instructions (9). All sample processing occurred in a biosafety cabinet with operators wearing gloves and other appropriate personal protective equipment. One sample was processed at a time, and the cleaning of work areas was done in accordance with the manufacturer's instructions (9). The FilmArray RP2 test consists of automated nucleic acid extraction, reverse transcription, nucleic acid amplification, and result analysis in approximately 45 min per run (i.e., per specimen). The FilmArray software performs automated result analysis, with each target in a valid run reported as “detected” or “not detected.” If either internal control fails, the software automatically provides a result of “invalid” for all panel analytes. There are 22 targets, as shown in Table 1, two of which are new to the FilmArray RP2. This study was conducted with an investigative-use-only (IUO) version of the FilmArray RP2 that is identical to the final FDA-cleared and CE-marked version. It is important to note that results for MERS-CoV are reported in this paper but are available only for the FilmArray RP2plus version of the product.

**TABLE 1:** Analytes detected by the FilmArray RP2

Analyte	Change relative to RP^*a*^
Viruses	
Adenovirus	Updated primers^*b*^, additional assays
Coronavirus 229E	Updated primers
Coronavirus HKU1	Not modified
Coronavirus NL63	Not modified
Coronavirus OC43	Updated primers
Human metapneumovirus	Updated primers
Human rhinovirus/enterovirus	Updated primers
Influenza A virus	Updated primers
H1	Updated primers
H1-2009	Not modified
H3	Updated primers
Influenza B virus	Not modified
Middle East respiratory syndrome coronavirus (MERS-CoV)	New
Parainfluenza virus 1	Updated primers
Parainfluenza virus 2	Updated primers
Parainfluenza virus 3	Updated primers
Parainfluenza virus 4	Updated primers
Respiratory syncytial virus	Updated primers
Bacteria	
Bordetella parapertussis (IS*1001*)	New
Bordetella pertussis (*ptxP*)	Not modified
Chlamydia pneumoniae	Not modified
Mycoplasma pneumoniae	Updated primers

a General pouch chemistry improvements led to increased sensitivity overall.

b Assay modified for broader inclusivity.

### Comparator testing.

Comparator testing consisted of SOC FilmArray RP testing performed at the source laboratory for all analytes in common between the FilmArray RP and the FilmArray RP2 (all analytes except MERS-CoV and B. parapertussis). All specimens were assumed negative for MERS-CoV, as it was not circulating in the United States during the time of enrollment for the study.

For B. parapertussis, two PCR assays targeting IS*1001* (the same target identified by the FilmArray RP2), followed by bidirectional sequencing, were used as the comparator method. Nucleic acid was extracted from specimens using MagNA Pure LC Total Nucleic Acid Kit–High Performance (Roche Diagnostics, Indianapolis, IN). Both real-time PCR comparator assays were validated and found to have a limit of detection (LoD) that was equivalent to that of the FilmArray RP2 assay. Testing was performed at BioFire in a blind manner. Comparator assay results were considered positive only when a bidirectional sequencing result of adequate quality was found to match a sequence for the expected analyte with an E value of 1.0E−30 or lower in the GenBank nucleotide database (Basic Local Alignment Search Tool [BLASTn] with default settings). A specimen was considered to be “positive” with a sequence-confirmed result from either assay.

### Results and discrepant analysis.

A FilmArray RP2 result was considered a true positive (TP) or true negative (TN) only when it agreed with the result from the comparator method. Discrepant analysis ensued when results were discordant, i.e., false-positive (FP) or false-negative (FN) results. When sufficient specimen volume was available, discordant specimens were investigated using a combination of retesting with the FilmArray RP2 or comparator methods, as well as testing with additional, independent molecular assays. For additional analysis of adenovirus targets, specimens were also tested with a combination of PCR assays targeting the DBP, penton, and *pol* genes (combined with bidirectional sequence analysis) (9) and the results of standard-of-care testing at one of the study sites (NCH) using an adenovirus laboratory-developed test (LDT) PCR targeting the hexon gene as described previously (10–12). Note that the performance data for positive percent agreement (PPA) and negative percent agreement (NPA) presented in this paper consist of unresolved data as presented in the package insert for the FDA-cleared test; discrepancy investigation is provided but was not used to recalculate performance data.

### Statistical analysis.

The exact binomial two-sided 95% confidence intervals (95% CI) were calculated for performance measures according to the Wilson score method.

## RESULTS

### Demographics.

A total of 1,612 prospective study specimens collected from geographically/demographically diverse subject populations were analyzed in this study. Overall, the study included specimens from more male than female subjects (54% [867/1,612] and 46% [745/1,612], respectively). Most specimens were from pediatric subjects: 55% of the specimens were from children aged 5 years and under, 21% were from those aged 6 to 21 years, 17% were from adults over the age of 50 years, and 8% were from adults aged 22 to 49 years. The majority of the specimens were obtained from hospitalized subjects and those visiting the emergency department (40% [640/1,612] and 40% [643/1,612], respectively), and 20% were obtained from subjects seen in an outpatient setting (329/1,612).

### FilmArray RP2 test performance.

A total of 1,623 specimens met the inclusion criteria and were initially tested in the clinical evaluation. The overall success rate for the initial test of these specimens was 99.3% (1,611/1,623); 12 tests were unsuccessful (1 due to an incomplete test, 1 due to an instrument error, and 10 due to control failures). Eleven of these specimens were successfully retested. In addition, another 10 specimens were later excluded for protocol reasons, resulting in a total of 1,612 specimens included in the data analysis.

### Summary of FilmArray RP2 findings.

The FilmArray RP2 detected at least one analyte in 1,020 of the 1,612 specimens tested, yielding an overall positivity rate of 63.3%, as shown in Table 2. The highest detection rate was seen in young children (≤5 years of age). The relative prevalence of each analyte among the positive specimens detected by the FilmArray RP2 is presented in Table 3. The most prevalent organisms detected during this study were HRV/EV, RSV, adenovirus, and FluA, which were found in 502 (31.1%), 199 (12.3%), 118 (7.3%), and 81 (5.0%) specimens, respectively. If taken together, coronaviruses (CoV-229E, -HKU1, -NL63, and -OC43) were the third most prevalent target, with 159 (9.9%) detections. For FluA H1 and the MERS-CoV targets, no positive analyte detections occurred in this prospective sample set. All other analytes were detected in fewer than 79 (<4.9%) specimens.

**TABLE 2:** Positivity rate for FilmArray RP2 for all samples and by age groups

Sample type/result	No. of samples	% of total
All samples		
Negative samples	592	36.7
Positive samples	1,020	63.3
Single detections	775	48.1
Codetections	245	15.2
Positive samples by age group		
≤5 yrs (*n* = 885)	698	78.9
6–21 yrs (*n* = 331)	196	59.2
22–49 yrs (*n* = 128)	53	41.4
50+ yrs (*n* = 268)	73	27.2

**TABLE 3:** Prevalence of FilmArray RP2-detected analytes stratified by age group

Analyte	Prevalence of analyte in indicated subject group									
	Overall (*n* = 1,612)		≤5 yrs (*n* = 885)		6–21 yrs (*n* = 331)		22–49 yrs (*n* = 128)		≥50 yrs (*n* = 268)	
	No.	%	No.	%	No.	%	No.	%	No.	%
Viruses										
Adenovirus	118	7.3	96	10.8	18	5.4	2	1.6	2	0.7
Coronavirus 229E	16	1.0	3	0.3	7	2.1	1	0.8	5	1.9
Coronavirus HKU1	55	3.4	37	4.2	9	2.7	2	1.6	7	2.6
Coronavirus NL63	50	3.1	41	4.6	6	1.8	2	1.6	1	0.4
Coronavirus OC43	38	2.4	28	3.2	7	2.1	0	0	3	1.1
Human metapneumovirus	81	5.0	60	6.8	12	3.6	3	2.3	6	2.2
Human rhinovirus/enterovirus	502	31.1	379	42.8	88	26.6	16	12.5	19	7.1
Influenza virus A	78	4.8	29	3.3	20	6.0	13	10.2	16	6.0
H1	0	0	0	0	0	0	0	0	0	0
H1-2009	74	4.6	26	2.9	19	5.7	13	10.2	16	6.0
H3	4	0.2	3	0.3	1	0.3	0	0	0	0
Influenza B	16	1.0	7	0.8	7	2.1	1	0.8	1	0.4
Middle East respiratory syndrome coronavirus (MERS-CoV)	0	0	0	0	0	0	0	0	0	0
Parainfluenza virus 1	10	0.6	9	1.0	0	0	1	0.8	0	0
Parainfluenza virus 2	54	3.3	39	4.4	10	3.0	1	0.8	4	1.5
Parainfluenza virus 3	53	3.3	44	5.0	6	1.8	2	1.6	1	0.4
Parainfluenza virus 4	16	1.0	13	1.5	1	0.3	0	0	2	0.7
Respiratory syncytial virus	199	12.3	168	19.0	10	3.0	8	6.3	13	4.9
Bacteria										
Bordetella parapertussis (IS*1001*)	6	0.4	4	0.5	2	0.6	0	0	0	0
Bordetella pertussis (*ptxP*)	3	0.2	0	0	3	0.9	0	0	0	0
Chlamydia pneumoniae	6	0.4	1	0.1	4	1.2	1	0.8	0	0
Mycoplasma pneumoniae	28	1.7	10	1.1	14	4.2	3	2.3	1	0.4

A summary of performance characteristics for individual FilmArray RP2 targets is presented in Table 4. PPA and NPA were calculated with respect to the comparator methods along with 95% CI. The FilmArray RP2 demonstrated a PPA of 91.7% or greater for all but three analytes. Nine of 22 analytes demonstrated a PPA of 100%: CoV-HKU1, CoV-NL63, FluA, FluA H1-2009, FluA H3, FluB, PIV1, PIV4, and C. pneumoniae. Eight other targets demonstrated a PPA of <100% but ≥90.0%: adenovirus, CoV-229E, hMPV, HRV/EV, PIV2, PIV3, RSV, and M. pneumoniae. For FluA H1 and MERS-CoV, no PPA could be calculated. The three analytes demonstrating a PPA of <90.0% were CoV-OC43 (80.5%), B. parapertussis (85.7%), and B. pertussis (66.7%). Additionally, nine analytes demonstrated a lower bound of the two-sided 95% CI of <80.0% due to few or no observations in the study. Overall, the FilmArray RP2 demonstrated an NPA of ≥93.5% for all analytes, with lower bounds of the two-sided 95% CI of ≥91.9%.

**TABLE 4:** Performance summary and characteristics of FilmArray RP2 versus those of the comparator assays^*a*^

Analyte	PPA^*b*^			NPA		
	TP/(TP + FN)	%	95% CI	TN/(TN + FP)	%	95% CI
Viruses						
Adenovirus	70/74	94.6	86.9–97.9	1,490/1,538	96.9	95.9–97.6
Coronavirus 229E	11/12	91.7	64.6–98.5	1,595/1,600	99.7	99.3–99.9
Coronavirus HKU1	43/43	100	91.8–100	1,557/1,569	99.2	98.7–99.6
Coronavirus NL63	40/40	100	91.2–100	1,562/1,572	99.4	98.8–99.7
Coronavirus OC43	33/41	80.5	66.0–89.8	1,566/1,571	99.7	99.3–99.9
Human metapneumovirus	73/75	97.3	90.8–99.3	1,529/1,537	99.5	99.0–99.7
Human rhinovirus/enterovirus	425/436	97.5	95.5–98.6	1,099/1,176	93.5	91.9–94.7
Influenza virus A	78/78	100	95.3–100	1,531/1,531	100	99.7–100
H1	0/0			1,609/1,609	100	99.8–100
H1-2009	74/74	100	95.1–100	1,535/1,535	100	99.8–100
H3	4/4	100	51.0–100	1,605/1,605	100	99.8–100
Influenza virus B	14/14	100	78.5–100	1,596/1,598	99.9	99.5–100
Middle East respiratory syndrome coronavirus (MERS-CoV)	0/0			1,612/1,612	100	99.8–100
Parainfluenza virus 1	9/9	100	70.1–100	1,602/1,603	99.9	99.6–100
Parainfluenza virus 2	46/47	97.9	88.9–99.6	1,557/1,565	99.5	99.0–99.7
Parainfluenza virus 3	43/45	95.6	85.2–98.8	1,557/1,567	99.4	98.8–99.7
Parainfluenza virus 4	9/9	100	70.1–100	1,596/1,603	99.6	99.1–99.8
Respiratory syncytial virus	175/176	99.4	96.9–99.9	1,412/1,436	98.3	97.5–98.9
Bacteria						
Bordetella parapertussis (IS*1001*)	6/7	85.7	48.7–97.4	1,605/1,605	100	99.8–100
Bordetella pertussis (*ptxP*)	2/3	66.7	20.8–93.9	1,608/1,609	99.9	99.6–100
Chlamydia pneumoniae	5/5	100	56.6–100	1,606/1,607	99.9	99.6–100
Mycoplasma pneumoniae	23/24	95.8	79.8–99.3	1,583/1,588	99.7	99.3–99.9

a These data are presented based on a comparator assay only and do not reflect any discordant analysis.

b The terms PPA (positive percent agreement) and NPA (negative percent agreement) are used instead of sensitivity and specificity to indicate that a non-gold standard comparator (e.g., PCR) was used for the analysis.

### Comparator analysis and discrepancy investigation.

There were a total of 33,843 analyzable FilmArray RP2 organism results for the 1,612 specimens. The overall percent agreement between the FilmArray RP2 and the comparator testing was 99.2% (33,586/33,843). There were 1,329 detected organism results with the FilmArray RP2; the comparator methods were positive for 1,138 analytes. The overall PPA with respect to the comparator method was 97.1% (1,105/1,138). There were 32,481 results not detected with the FilmArray RP2; the comparator methods were negative for 32,705 analytes. The overall NPA with respect to the comparator method was 99.3% (32,481/32,705).

Using comparator testing results as the truth, there were 224 FP detections and 33 FN detections overall; additional discrepancy analysis was performed for these 257 samples. For the 114 FP cases (51%) and the 14 FN cases (42%), there was supportive evidence for the FilmArray RP2 result, bringing the adjudicated overall concordance for the positive and negative results to 98.5% and 99.7%, respectively. A summary of the discrepancy investigation is presented in Table 5.

**TABLE 5:** Results of discrepant investigation for FilmArray RP2

Analyte	FN^*a*^			FP		
	Original result (total)	Discrepant investigation outcome^*b*^		Original result (total)	Discrepant investigation outcome	
		RP2 confirmed (TN)	RP2 unconfirmed (FN)		RP2 confirmed (TP)	RP2 unconfirmed (FP)
Viruses						
Adenovirus	4	1	3	48	40	8
Coronavirus 229E	1	1	0	5	0	5
Coronavirus HKU1	0			12	3	9
Coronavirus NL63	0			10	3	7
Coronavirus OC43	8	2	6^*c*^	5	2	3
Human metapneumovirus	2	2	0	8	6	2
Human rhinovirus/enterovirus	11	6	5	77	33	44
Influenza virus A	0			0		
H1	0			0		
H1-2009	0			0		
H3	0			0		
Influenza virus B	0			2	2	0
Middle East respiratory syndrome coronavirus (MERS-CoV)	0			0		
Parainfluenza virus 1	0			1	0	1
Parainfluenza virus 2	1	1	0	8	5	3
Parainfluenza virus 3	2	0	2	10	4	6
Parainfluenza virus 4	0			7	1	6
Respiratory syncytial virus	1	1	0	24	8	16
Bacteria						
Bordetella parapertussis (IS*1001*)	1	0	1	0		
Bordetella pertussis (*ptxP*)	1	0	1	1	1	0
Chlamydia pneumoniae	0			1	1	0
Mycoplasma pneumoniae	1	0	1	5	5	0
Total	33	14	19	224	114	110

a Result disposition based on initial testing versus comparator.

b RP2 confirmed, the results of discrepant analysis supported the original FilmArray RP2 result as true negative (TN) or true positive (TP). RP2 unconfirmed, the results of discrepant analysis did not support the original FilmArray RP2 result, and the result was considered false negative (FN) or false positive (FP).

c Six FN specimens were all TP for coronavirus HKU1 due to a known cross-reactivity in the comparator method (9).

For the viral analytes, the FilmArray RP2 detected a total of 1,286 viral analytes. Using the comparator results as the truth, the overall PPA and NPA are 97.3% (1,069/1,099) and 99.2% (26,079/26,296), respectively. The results for several analytes of significant interest are further detailed below.

For adenovirus, a significant increase in detections was observed in comparison to those by the FilmArray RP, with a total of 118 detections, of which 48 (40.7%) were FP. FP specimens with sufficient volume were retested with the FilmArray RP to see if the original result had been an anomaly. When possible, specimens were also tested with a combination of PCR/sequencing assays targeting the DBP (*n* = 38), penton (*n* = 25), and *pol* (*n* = 16) genes and the results of the NCH LDT assay (*n* = 11). Combined, these investigations found additional evidence of adenovirus presence in 40 of the 48 FP specimens (Table 6). All 40 of these specimens had late amplification on the FilmArray RP2 test, suggestive of low levels of analyte in these specimens. The four FP specimens for which the FilmArray RP retest was positive also had late amplification, suggestive of a low level of analyte.

**TABLE 6:** Summary of species determinations for all adenovirus-positive samples

Adenovirus species	Original RP2 result characterization compared to that of RP^*a*^		
	No. of TP	No. of FN	No. of FP^*b*^
A	0	0	2
B	20	0	7
C	47	3	17^*c*^
D	0	0	1
E	0	0	0
F	0	0	11^*c*^
Unable to determine species	3	1	11
Total	70	4	48

a TP, true positives = positive with RP and RP2; FN, false negatives = RP positive, RP2 negative; FP, false positives = RP negative, RP2 positive.

b For specimens yielding a species identification (*n* = 40), adenovirus was considered confirmed (3 FN missed by RP2, and 37 FP missed by RP).

c One specimen indicated a coinfection with adenovirus species C and F.

There were also 4 FN results for adenovirus. Additional discrepant analysis for these specimens included retesting with the FilmArray RP2, a combination of PCR assays as described above, and any available NCH LDT results for adenovirus. Combined, these investigations found additional evidence of adenovirus presence in three of the four FN specimens. Analysis of the FN specimen for which the FilmArray RP2 retest was positive indicated late amplification, suggesting low analyte levels. All FN were adenovirus species C based on sequence analysis. A comprehensive summary of the adenovirus discordant analysis is provided in Table 6.

Among the coronaviruses, all but one of the four targets demonstrated good performance, with a PPA of ≥91% and an NPA of ≥99.1%. The exception was CoV-OC43, which demonstrated a PPA of 80.5%. The majority of FN specimens observed were due to a known cross-reactivity in the comparator method (see BioFire FilmArray RP package insert at https://www.online-ifu.com/ITI0040): a FilmArray RP-detected result for coronavirus OC43 due to cross-reactivity with CoV-HKU1 is suspected whenever the FilmArray RP reports detections for both CoV-HKU1 and CoV-OC43. This cross-reactivity has been corrected by the redesign of the CoV-OC43 assay for the FilmArray RP2. Six of eight FilmArray RP2 FN specimens were TP for CoV-HKU1, i.e., codetections reported by the FilmArray RP and suggestive of this known cross-reactivity. As stated previously, no MERS-CoV was detected in the cohort. Of note is an NPA of 100%, indicating a lack of cross-reactivity with other coronaviruses. Data for some archived MERS-CoV specimens and contrived MERS-CoV samples are provided in the manufacturer's package insert for the FilmArray RP2*plus* (9).

The FluA targets showed no FP or FN detections; however, there were no positive detections for FluA H1 during the study period, which was predominated by FluA H1-2009. For FluB, there were two FP detections, which were confirmed on further investigation as TP. FluA, FluA H1, FluA H1-2009, and FluA H3 results were excluded from analyses for three specimens due to initial results of “influenza A equivocal” or “influenza A no subtype detected” by either FilmArray RP2 or FilmArray RP testing and insufficient specimen volume for retesting.

Detections for HRV/EV were numerous, with a total of 502, the highest of all detections in the trial. There were 77 FP results and 11 FN results. Specimens with sufficient volume were retested with the FilmArray RP or FilmArray RP2. When possible, specimens were also tested with a combination of five PCR assays targeting the 5′ UTR gene. For the FP samples, 29 were positive with a FilmArray RP retest; late amplification for 28 of the 29 specimens was suggestive of low levels of analyte. Four more were positive with PCR assays. For the FN samples, four specimens were positive with the FilmArray RP2 on repeat testing and one was positive with PCR assays. Three of the four FN specimens for which the FilmArray RP2 retest was positive had late amplification, suggestive of low levels of analyte.

RSV detections totaled 199, making it the second most common analyte. Eight of 24 FP specimens were observed to contain RSV by independent molecular methods or retesting with the FilmArray RP. These may have been missed by the SOC FilmArray RP test due to an estimated hundredfold difference in LoD between the FilmArray RP and FilmArray RP2 (9).

Using the comparator results as the truth, the overall PPA and NPA are 92.3% (36/39) and 99.9% (6,402/6,409), respectively, for all bacterial targets. The number of detections for each bacterial target was low (≤6), with the exception of that for M. pneumoniae (*n* = 28) (Table 4). The two bacterial analytes demonstrating a PPA of <90.0% were both low prevalence: B. parapertussis (*n* = 6), and B. pertussis (*n* = 3).

The bacterial targets tended to be single analyte detections (B. pertussis, 3/3; C. pneumoniae, 5/6; and M. pneumoniae, 21/28) with no copathogen present. For B. parapertussis, all six detections were in the context of a codetection with one or more viruses. No sample had two bacterial targets detected. Discordant analysis for the bacterial targets in shown in Table 5.

## DISCUSSION

This study of the FilmArray RP2 demonstrated the performance of the test in a large prospective study of 1,612 residual NPS samples, with 33,843 results generated. These data are significant, as this is a substantial change from the results with RP, and the test will be adopted for use in a large number of clinical laboratories. The number of positive detections was relatively high for most organisms; notable exceptions were the results for MERS-CoV and FluA H1, which were not circulating in the study populations during the study period. The FilmArray RP2 testing system was shown to be reliable, with very few failures (99.3% success on the initial test attempt), and rapid, with results available in approximately 45 min, which is shorter than that of the FilmArray RP (approximately 65 min run time). The data presented here along with testing of archived positive NPS in VTM specimens and contrived specimens (not shown) (9) were used as part of the regulatory submissions for the FilmArray RP2 and RP2*plus*, which received 510(k) clearance in the United States (RP2) and CE/IVD marking in the European Union (RP2*plus*) in June 2017. The FilmArray RP2*plus* received *de novo* clearance in the United States in November 2017.

Periodically updating testing that has been implemented is an important concept. The College of American Pathologists covers this for laboratory-developed testing in its *Microbiology Checklist*, stating that laboratories should have written policies and procedures to evaluate nucleic acid tests for compatibility with currently circulating microbial strains (13). For testing cleared by the FDA, The FilmArray RP2 represents the fourth iteration of the multiplex panel since its introduction in 2011, providing an update of the primer probes based on a reexamination of known sequences for the majority of the pathogens and adjustment of the assay conditions to maximize performance. As noted, there were a significant number of detections by RP2 that were not found by RP (*n* = 224). The overall design goal for RP2 was to increase the sensitivity for all analytes relative to that of RP, and this may account for a significant number of the observed FP detections. This is supported by LoD studies reported in the product inserts (9, 14) and the discordant analysis performed in this study. The increased inclusivity/sensitivity and decreased time to result to 45 min for the FilmArray RP2 may lead to improvements in outcomes, such as length of stay or proper stewardship, and warrant further study.

Viruses are a common cause of upper respiratory infections in both adult and pediatric populations, and this was also seen in our study cohort. Viral detections were notably higher than those of the bacterial targets (1,286 viral detections versus 43 bacterial detections). The FilmArray RP2 showed an increased positive detection rate for all viral targets in comparison to that of the FilmArray RP (217 more detections, with 107 supported by additional discrepancy investigation), with the exception of coronavirus OC43 (Table 5), reflecting the increased sensitivity and inclusivity of the FilmArray RP2.

The most common viral analyte was HRV/EV, with a total of 502 detections versus 436 detections with the FilmArray RP. The increased number of HRV/EV detections may or may not be associated with true disease causation, as the majority (61.2% [see Table S1 in the supplemental material]) were in the context of codetection with other viral targets. Rhinovirus has been reported as a commonly detected target among asymptomatic individuals, with rates ranging from 8 to 50% depending on the study (15–17). While the FilmArray RP2 was updated to broaden inclusivity, there was no change in specificity for HRV/EV, so there are still cross-reactions with enteroviruses and hence the rhinovirus/enterovirus designation.

One of the more extensive modifications occurred in the detection of adenovirus. Previous studies by Leber et al. demonstrated a lack of sensitivity with the FilmArray RP for adenovirus types A, D, and F (12) despite an earlier redesign of the FilmArray RP in 2013 (18). The redesign of the FilmArray RP2 specifically targeted all genotypes to include genotypes A to F and not only those typically associated with respiratory infections (types B, C, and E). In our cohort, genotypes A, B, C, D, and F were demonstrated to be detected by the FilmArray RP2. Detection of all genotypes is important, particularly in the immunocompromised, where the finding of adenovirus of any genotype in the NPS in VTM may precede systemic infections (11, 12). In addition, the identification of species F in respiratory specimens has been reported in patients with respiratory illness (19) as well as in 2.3% of pediatric patient samples obtained after routine adenoidectomy/tonsillectomy (20).

Overall, there were relatively low numbers of bacterial detections with the FilmArray RP2 (*n* = 43). The reasons for this are likely due to true disease prevalence differences during the study period. Also, as seen in our data (Table S1), the codetection of bacteria and viruses is not common, particularly with B. pertussis, as has been previously reported (21, 22). The target gene for B. pertussis in both the FilmArray RP and the FilmArray RP2 is the toxin promoter region. This single-copy gene is known to be more specific than the more commonly used insertion sequence 481 (IS*481*) gene that is a multicopy target present in several Bordetella species. While having greater specificity, the toxin gene target may be less sensitive, as has been reported previously (23). The diagnosis of pertussis-like illness is improved with the inclusion of the insertion sequence element 1001 target for B. parapertussis in the FilmArray RP2. B. parapertussis is known to cause a pertussis-like illness and can cocirculate with other Bordetella species (24, 25). The prevalence of B. parapertussis is uncertain, as it is not a reportable disease like B. pertussis and is not tested for as commonly (26, 27). M. pneumoniae was the most common of the bacterial analytes, with 28 detections, more than with the FilmArray RP. However, it should be noted that use of an NPS specimen for the detection of M. pneumoniae may be suboptimal, particularly when diagnosing lower respiratory tract infection (28, 29).

Overall, the percentage of discrepant results was low (0.76%, *n* = 257) (Table 5), suggesting that the previous version of the FilmArray RP had relatively robust performance. Discrepancy analysis using FilmArray RP retests and PCR and bidirectional sequencing confirmed 114 of 224 FP (51%), which is strong evidence that the FilmArray RP2 has increased sensitivity compared to the FilmArray RP. There are some limitations of this study. This was a prospective study; however, some samples were frozen at −70°C or less prior to testing. However, data indicated that the frozen storage did not significantly affect performance (9). The study period bridges one calendar year (2016) and includes only two partial respiratory seasons, so variations in circulating strains, particularly FluA, are limited. The comparator method for 20 of the targets was the FilmArray RP. Data concerning FilmArray RP2 performance compared to that of other amplified platforms or culture are not provided and will await other studies. Finally, the lack of detection of MERS-CoV and FluA H1 in the prospective study limited the data on the performance for these targets.

A significant redesign of the FilmArray RP2 has demonstrated excellent sensitivity and specificity in this multicenter clinical trial. This is an important step both for individual improvements in pathogen detection and as recognition by the manufacturer that continuous improvements in monitoring and inclusion of new or emerging strains or species are important. Both have been incorporated into the design of the FilmArray RP2, improving its performance for the detection of infectious agents that are involved in respiratory infections. These changes include new targets (MERS-CoV and B. parapertussis), improvements to existing targets, and a decreased time to result. These improvements, combined with the simplicity of the testing process and a shorter time to result, make the FilmArray RP2 a significant improvement in diagnostic testing.

## Supplementary Material

Supplemental material
