# Supplementary material for: Multicenter Evaluation of BioFire FilmArray Respiratory Panel 2 for Detection of Viruses and Bacteria in Nasopharyngeal Swab Samples
Source: J Clin Microbiol. 2018 May 25;56(6):e01945-17. doi: 10.1128/JCM.01945-17 (PMC5971546; doi:10.1128/JCM.01945-17)
Supplement: Supplemental material [file supp_56_6_e01945-17__index.html]

Supplemental material 

# Multicenter Evaluation of BioFire FilmArray Respiratory Panel 2 for Detection of Viruses and Bacteria in Nasopharyngeal Swab Samples

## Supplemental material

- Supplemental file 1 -

  Table S1 (Prevalence of analytes in codetections as determined by FilmArray RP2)

  PDF, 15K
