# Supplementary material for: Multicenter Evaluation of BioFire FilmArray Respiratory Panel 2 for Detection of Viruses and Bacteria in Nasopharyngeal Swab Samples
Source: J Clin Microbiol. 2018 May 25;56(6):e01945-17. doi: 10.1128/JCM.01945-17 (PMC5971546; doi:10.1128/JCM.01945-17)
Supplement: Supplemental material [file JCM.01945-17_zjm999095961s1.pdf]

SUPPLEMENTAL TABLES:

SUPPLEMENTAL TABLE 1 Prevalence of Analytes in Co Detections as determined by the FilmArray RP2

Analyte	Prevalence in Co-Detections (N=245)	
Viruses		
Adenovirus	85	34.7%
CoV-229E	6	2.4%
CoV-HKU1	41	16.7%
CoV-NL63	31	12.7%
CoV-OC43	19	7.8%
hMPV	33	13.5%
HRV/EV	150	61.2%
FluA H1	0	0%
FluA H1-2009	9	3.7%
FluA H3	2	0.8%
FluB	6	2.4%
MERS-CoV	0	0%
PIV1	5	2.0%
PIV2	15	6.1%
PIV3	21	8.6%
PIV4	12	4.9%
RSV	105	42.9%
Bacteria		
B. parapertussis (IS*1001*)	6	2.4%
B. pertussis (*ptxP*)	0	0%
C. pneumoniae	1	0.4%
M. pneumoniae	7	2.9%
